# Mr. Woodham in Answer to Mr. Walker

**Published:** 1816-11

**Authors:** 


*For the London Medical and Physical Journal.*

### Mr. Woodham *in Answer to Mr. Walker.*

INSTEAD of repelling the charges of filling your Journal with common-place, and attempting to undermine vaccination, Mr. Walker has chosen to prefer against me three accusations; and, if to express strongly that which we feel strongly be deemed asperity, and a quotation, not, I trust, inapposite, be considered as pedantry, of two of them I acknowledge myself guilty; but I must beg leave to plead *not guilty* to the third;—for, if Mr. W. will take the trouble (no other person, I fear, will) to compare his papers with mine, *he*, and not myself, will be found to be the *wordy* gentleman. Mr. W. desires I would not be surprised should he not notice any comments of mine on his future communications? I assure him I will not; neither will I, as he *requests* it (I should otherwise, I believe), attribute his silence to incompetency of answering; but, whatever I may think of want of excitement, he must excuse me from regarding want of leisure as a cause: *that* is forbidden, by his regular and "lengthy" communications to your impartial Journal.

*London; Oct.* 2, 1816.

